# Assessing the quality of electronic medical records in academic hospitals: A multi-center study in Iran

**DOI:** 10.3389/fdgth.2022.856010

**Published:** 2022-11-23

**Authors:** Hedieh Zabolinezhad, Saeid Eslami, Mohammad Reza Hassibian, Sara Dorri

**Affiliations:** ^1^ Department of Medical Informatics, School of Medicine, Mashhad University of Medical Sciences, Mashhad, Iran; ^2^ Health Information Technology Research Center, Isfahan University of Medical Sciences, Isfahan, Iran

**Keywords:** hospital information system, electronic medical records, electronic medical record quality, software HISRA, data completeness, data accuracy, data quality

## Abstract

**Objective:**

The present study aimed to assess the quality of electronic medical records (EMR) retrieved from hospital information systems (HIS) of three educational hospitals in Mashhad, Iran.

**Methods:**

In this multi-center, cross-sectional study, inpatient electronic records collected from three academic hospitals were categorized into five data groups, namely demographics (D); care handler (CH), indicating the doers of the medical actions; diagnosis and treatment (DT); administrative and financial (AF); and laboratory and Para clinic (LP). Next, we asked 25 physicians from the three academic hospitals to determine data elements of medical research and education value (called research and educational data) in every group. Flowingly, the quality of the five data groups (completeness * accuracy) was reported for entire sampled data and those specified as research and educational data, based on the exact concordance between electronic medical records and corresponding paper records. HISRA, standing for HIS recording ability, was also assessed compared to data elements of standard paper forms.

**Results:**

For entire data, HISRA was 58.5%. In all hospitals, the highest data quality (more than 90%) belongs to D and AF data groups, and the lowest quality goes to CH and DT groups (less than 50%, and 60%, respectively). For research and educational data, HISRA was 47%, and the quality of D and AF data groups were the highest (nearly 100%), while CH and DT stood around 50% and 60% in order. The quality of the LP data group was almost 85% in all hospitals but hospital C (well over 30%). Total data quality for the hospitals was almost less than 70%.

**Conclusions:**

The low quality of electronic medical records was mostly a result of incompleteness, while the accuracy was relatively good. Results showed that the HIS application development mainly focused on administrative and financial aspects rather than academic and clinical goals.

## Introduction

A hospital information system (HIS) is an integrated, computer-assisted system that commonly reflects all hospital operations dimensions. It is used to store and retrieve information related to clinical, administrative, financial, and legal tasks to meet the needs of all authorized system users in the hospital (1). HIS is a potentially rich source usually used for research and educational purposes besides the quality of care improvement and managerial activities. A HIS with high-quality data can improve the quality of health care, monitor patient condition, and therapeutic response, reduce the frequency of errors, avoid adverse drug interactions, and reduce hospital costs (2). In recent years, there has been a growing interest in researching electronic medical records (EMRs) collected in HIS (3). Medical studies, epidemiological investigations, and analysis of disease progression are conducted based on EMRs collected from HIS. Furthermore, patients’ EMRs can be used to train medical students and increase their clinical knowledge (4).

The validity of medical research and epidemiological studies done using EMRs strongly relies on the quality of recorded data (5). If the quality of data and reports recorded in HIS are not sufficiently high, the investment return is not guaranteed, and the health care system will not achieve the predefined objectives. Besides, high-quality EMRs can be considered a major educational tool in health care education (6) as it offers acknowledged benefits to medical students in academic environments (7). Therefore, the quality of patient EMRs is considered highly important in any hospital and should be frequently assessed to determine defective processes (8).

So far, studies have been done on HIS in Iran as a developing country, mainly focused on user satisfaction, financial issues (hospital costs, for example), and managerial and administrative functions (9). To our knowledge, a few studies have been performed to assess the HIS data quality by this study method.

Therefore, this study assessed the quality of electronic medical records especially those that possess educational and research value in terms of completeness, accuracy, and quality (completeness * accuracy), in academic hospitals in Mashhad, Iran. We also present the hospital information system recording ability (HISRA; the potential of the system to record the five necessary data groups) of the HIS.

## Materials and methods

In this study, we assessed completeness and accuracy as the measures of data quality. We used “Data Source Agreement” as the strategy of assessment and paper medical records were regarded as the assessment “standard” to which the EMRs were compared (3).

### Study location and sampling

We selected three academic hospitals, a 920-bed general hospital (A), a 144-bed pediatric hospital (B), and a 60-bed ophthalmic hospital (C) in Mashhad, which is Iran's second-largest city with about 3 million inhabitants. In these three hospitals, data were mainly recorded in the form of hard copies and some electronic recordings were performed in parallel.

All hospitals use the same version of HIS designed in compliance with the international standard for the exchange of medical information (HL7). The HIS software was launched in 2001 in all hospitals belonging to Mashhad University of Medical Sciences. It is connected to the three ancillary subsystems (laboratory, pharmacy, and radiology) and to the databases including SNOMED, ICD.9.cm, ICD.10, and California tariff that feed patients' EMR. Furthermore, the system records some clinical documentation including medication administration, physician orders, and clinical and surgical procedures.

The study was conducted within 90 days, from September 2019 through November 2019. Over this period, 150, 100, and 50 cases were selected in the three hospitals, A, B, and C, respectively. We used stratified random sampling as the number of selected cases in each hospital was proportionate to that hospital's daily inpatient discharges. We included only inpatient discharges for them both paper and electronic records were available.

We carried out a retrospective comparison between a sample of electronic records and hard copies to examine the quality of electronic medical records.

### Categorization of data elements and evaluation of HISRA

To collect data, we considered a checklist of entire data elements of a standard paper record. In the checklist, the data elements were categorized into five groups, namely demographics (D), care handler (CH); diagnosis and treatment (DT); administrative and financial (AF); and laboratory and Para clinic (LP).

HISRA, standing for Hospital information software recording ability, indicates the potential of the hospital information software to capture all data values from the paper records. For each data group, it was calculated as the ratio of the number of available data fields that can be filled in both paper charts and the HIS software, to the total number of data elements, within the paper charts.

### Specification of research and educational data elements

To specify data elements that serve medical research and educational objectives in particular, the checklist was sent to 25 physicians from the studied hospitals, who were all faculty members of University of Medical Sciences, Mashhad, Iran; they were asked to determine whether a given data element is “Necessary “or “Not necessary” concerning research and educational importance. Then, the content validity ratio (CVR) was calculated for each data element according to the collected answers. For a group size of 25 (the number of physicians included in this experiment), the critical number and critical CVR were calculated as 18 and 0.44, respectively. The data elements with CVR ≥ 0.44 were selected as elements with research and educational importance and called “research and educational data” (10).

### Data collection, completeness, and accuracy

For every sampled discharge, a checklist was completed by comparing the electronic and paper medical records. In the checklist, the presence or absence of each entry in the paper and the electronic records was determined to evaluate data completeness. In addition, the exact concordance between the two values recorded for a specific element in electronic and paper medical records was considered to measure data accuracy.

#### Completeness

“Completeness” was defined as the existence of an entry for a given element in the electronic medical record that is present in the paper record, regardless of its accuracy. Completeness was calculated as the ratio of the number of data entries found in patients' electronic records to the total number of data entries that existed in patients' paper records.

#### Accuracy

“Accuracy” was defined as a strict concordance between electronic data value and the corresponding value in the paper record. Accuracy was calculated as the ratio of the number of accurate data values to the total number of data values (11, 12).

### Data analysis

Finally, for entire data together with the research and educational data, the quality of each data group was calculated by multiplying accuracy by completeness (11, 12). The hospitals were statistically compared using *P*-value (<0.05), derived from the Pearson Chi-square test, to examine whether hospitals' difference considering data completeness and accuracy is statistically significant or the difference is by chance.

## Results

### Data elements with research and educational importance

Based on their educational and research value, data elements were specified as “essential” and “not essential” by 25 physician selected from the three hospitals; then CVR was calculated for each data element. Table 1 shows the data elements that meet the criteria of CVR ≥ 0.44, and were selected as elements with research and educational importance. In this table, data elements of the same data group and the same CVR are shown together in a row.

**Table 1:** Content validity ratio (CVR) for research and educational data elements.

Data group	Data elements	The number of physicians who specified data elements (CVR)
Demographics (D)	Date & Place of birth	25 (1)
	Marital status, Sex	20 (0.6)
	Occupation, Religion	18 (0.44)
Care Handler (CH)	Surgeon	22 (0.76)
	Attending Physician, Admitting Physician	20 (0.6)
	Anesthetist	18 (0.44)
Diagnosis and Treatment (DT)	Former Admission Record, Chief Complaints, Primary Diagnosis, Final Diagnosis, External Cause Of Injury, Disease Progress, Operations & Other Procedures, Patient's Condition At The Time Of Discharge, Cause of Death, Time & Date of Death, Present Illness, Past Medical History, Current Drug Therapy, Allergy, Addiction, Family History, Physical Examinations, Object Of Consultation, Consultant Observation, Blood Pressure After Using Anesthesia drugs, Urea, Hb and Hct, Blood sugar, Blood group,RH, Pulse, Vital Signs Before Operation, Time Of last Urination Before Operation, Pre-Operation Drugs & Time Of The Use, Pre-Anesthesia Drugs Effects, Anesthesia Time, Patient's Status In The Beginning / At The End Of the Operation, Fluids, Symbols, Name Of Operation, Type Of Operation, Direction, Pre/Post Operation Diagnosis, Procedure & Findings, General Condition, Awakens, Vital Signs, Skin color/ Temperature, Local/ General Cyanosis, Intra Venous Fluid, Fluid Absorption, Fluid Excretion, Plasma, Blood, Ordered Drugs (Name, Dose, Type, Frequency), Information Of Drug Administration, Medical procedures, Observations, Considerations And Sign Of Nurse, Vital Signs Control, Balance Chart Information,	25 (1)
	Number Of Hospitalizations, Consultation Request, Blood Pressure On Admission, Type Of Anesthesia, Physician's orders, Diet, Composite Graphic Chart Information	22 (0.76)
	Time & Date Of Examination, Start/End Time Of Operation, Date Of Operation	20 (0.6)
	Time In/Exit, Fluid Balance, Oxygen, Artificial Respiration, Date & Time Of Control, Date & Time Of Procedure	19 (0.52)
	Number Of Consultations, Recommendation After Discharge, Treatment Progress, Date Of Anesthesia, Observations & Treatments	18 (0.44)
Laboratory and Para clinic (LP)	Laboratory & X- Rays (Results), Ordered Laboratory Test, Ordered Para clinic Procedures, Para clinic reports (Results)	25 (1)
	Date of Laboratory test, Date of Para clinic Procedure	22 (0.76)
Administrative and Financial (AF)	Operation & Other Procedures code	25 (1)
	Length of Stay	19 (0.52)
	Discharge Date & Time, Admission Date & Time	18 (0.44)

Bold values are percent.

### HISRA of the HIS software

Concerning entire data elements, HISRA was 58.5%, while it was 47% for research and educational data elements (Tables 2, 3). Among entire data elements, HISRA of CH, and DT groups (75% and 43%, respectively) were lower than those of D, LP and AF groups (100%, 100%, and 91%, respectively).

**Table 2:** HISRA of the HIS concerning entire data elements.

Data group	HISRA
D	12 out of 12 **(100%)**
CH	24 out of 32 **(75%)**
DT	65 out of 151 **(43%)**
LP	7 out of 7 **(100%)**
AF	29 out of 32 **(91%)**
Total	137 out of 234 **(58.5%)**

D, Demographics; CH, Care Handler; DT, Diagnosis and Treatment; AF, Administrative and Financial; LP, Laboratory and Para clinic. Bold values are percent.

**Table 3:** HISRA of the HIS concerning research and educational data elements.

Data group	HISRA
D	6 out of 6 **(100%)**
CH	3 out of 4 **(75%)**
DT	51 out of 130 **(39%)**
LP	6 out of 6 **(100%)**
AF	6 out of 6 **(100%)**
Total	72 out of 152 **(47%)**

D, Demographics; CH, Care Handler; DT, Diagnosis and Treatment; AF, Administrative and Financial; LP, Laboratory and Para clinic. Bold values are percent.

Similarly, for research and educational data elements, HISRA of CH, and DT groups (75% and 39%, in order) were far lower than those of D, LP and AF groups (100%).

### Data quality in hospitals A, B and C

In total, 300 discharged cases were assessed from September 2020 to November 2020. Table 4 shows the completeness, accuracy, and quality of five data groups in the three hospitals for entire data elements. Similarly, Table 5 shows them for research and educational data.

**Table 4:** Completeness, accuracy and quality of data groups concerning entire data elements.

	Data group	Completeness	Accuracy	Quality (Completeness * Accuracy)
Hospital A (*n* = 150)	D	1,480/1,528 **(97%)**	1,480/1,480 **(100%)**	**97%**
	CH	1,500/3,631 **(41%)**	1,159/1,500 **(77%)**	**32%**
	DT	6,163/12,080 **(51%)**	5,139/6,163 **(83.40%)**	**42%**
	LP	180/200 **(90%)**	164/180 **(91%)**	**82%**
	AF	3,608/3,858 **(93.5%)**	3,444/3,608 **(95%)**	**89%**
	Total	12,931/21,297 **(61%)**	11,386/12,931 **(88%)**	**54%**
Hospital B (*n* = 100)	D	790/800 **(99%)**	790/790 **(100%)**	**99%**
	CH	700/1,553 **(45%)**	697/700 **(97%)**	**44%**
	DT	3,300/5,204 **(63%)**	3,177/3,300 **(96%)**	**60.5%**
	LP	150/165 **(91%)**	150/150 **(100%)**	**91%**
	AF	2,203/2,258 **(97.5%)**	2,156/2,203 **(98%)**	**95.5%**
	Total	7,143/9,980 **(72%)**	6,952/7,143 **(97%)**	**70%**
Hospital C (*n* = 50)	D	458/461 **(99%)**	453/458 **(99%)**	**98%**
	CH	330/784 **(42%)**	323/330 **(98%)**	**41%**
	DT	1,784/2,467 **(72%)**	1,508/1,784 **(84.5%)**	**61%**
	LP	21/65 **(32%)**	20/21 **(95%)**	**30%**
	AF	1,125/1,147 **(98%)**	1,112/1,125 **(99%)**	**97%**
	Total	3,718/4,924 **(75.5%)**	3,416/3,718 **(92%)**	**69%**

D, Demographics; CH, Care Handler; DT, Diagnosis and Treatment; AF, Administrative and Financial; LP, Laboratory and Para clinic.

N is the number of assessed cases. Bold values are percent.

**Table 5:** Completeness, accuracy and quality of research and educational data elements.

	Data group	Completeness	Accuracy	Quality (Completeness*Accuracy)
Hospital A (*n* = 150)	D	769/769 **(100%)**	769/769 **(100%)**	**100%**
	CH	445/463 **(96%)**	340/445 **(70%)**	**67%**
	DT	5,930/10,364 **(57%)**	4,988/5,930 **(84%**)	**48%**
	LP	170/185 **(92%)**	164/170 **(96.5%)**	**89%**
	AF	67/82 **(82%)**	62/67 **(92.5%)**	**76%**
	Total	7,381/11,863 **(62%)**	6,323/7,381 **(86%)**	**53%**
Hospital B (*n* = 100)	D	311/312 **(100%)**	311/311 **(100%)**	**100%**
	CH	246/400 **(61.5%)**	238/246 **(97%)**	**59%**
	DT	3,110/4,350 **(71.5)**	2,980/3,110 **(96%)**	**69%**
	LP	150/158 **(95%)**	150/150 **(100%)**	**95%**
	AF	45/48 **(94%)**	43/45 **(96%)**	**90%**
	Total	3,862/5,268 **(73%)**	3,722/3,862 **(96%)**	**70%**
Hospital C (*n* = 50)	D	200/200 **(100%)**	200/200 **(100%)**	**100%**
	CH	48/140 **(34%)**	48/48 **(100%)**	**34%**
	DT	1,784/2,308 **(77%)**	1,508/1,784 **(84.5%)**	**65%**
	LP	21/62 **(34%)**	20/21 **(95%)**	**32%**
	AF	27/27 **(100%)**	27/27 **(100%)**	**100%**
	Total	2,080/2,737 **(76%)**	1,803/2,080 **(86.70%)**	**66%**

D, Demographics; CH, Care Handler; DT, Diagnosis and Treatment; AF, Administrative and Financial; LP, Laboratory and Para clinic.

N is the number of assessed cases. Bold values are percent.

Concerning entire data, the total data quality of hospital B (70%) is higher than hospitals A and C (54% and 69%), and the quality of the D data group (more than 97%) is considerably higher than the other groups in the three hospitals.

Concerning research and educational data elements, hospital B has better total data quality compared to the others (70% vs. 53 and 66%). Moreover, the D data group has the highest quality (100%) in the three hospitals. It is equal to the AF in hospital C and followed by the LP data group in hospitals B and A (95 and 89%, in order).

### Comparison of completeness and accuracy among the three hospitals

Table 6 shows the probability values of completeness and accuracy for entire data, which differ significantly among the three hospitals. The completeness of data groups but D were significantly different when comparing the three hospitals. There were significant differences in terms of accuracy of CH, DT, and LP data groups among the three hospitals (Table 7).

**Table 6:** Comparison of completeness and accuracy for entire data values.

	Completeness	Accuracy
	*P*-value	*P*-value
D	**<0.05**	**<0.05**
CH	**0.041**	**<0.05**
DT	**<0.05**	**<0.05**
LP	**<0.05**	**<0.05**
AF	**<0.05**	**<0.05**

D, Demographics; CH, Care Handler; DT, Diagnosis and Treatment; AF, Administrative and Financial; LP, Laboratory and Para clinic. Bold values are percent.

**Table 7:** Comparison of completeness and accuracy for research and educational data values.

	Completeness	Accuracy
	*P*-value	*P*-value
D	**0.211**	**>0.05**
CH	**<0.05**	**<0.05**
DT	**<0.05**	**<0.05**
LP	**<0.05**	**0.056**
AF	**0.013**	**0.318**

D, Demographics; CH, Care Handler; DT, Diagnosis and Treatment; AF, Administrative and Financial; LP, Laboratory and Para clinic. Bold values are percent.

## Discussion

We assessed the data quality of the HIS used in the hospitals belonging to the Mashhad University of Medical Sciences. To this, we performed a retrospective comparison between a sample of electronic and paper medical records in three academic hospitals. We calculated HISRA and five data groups' quality (completeness * accuracy), namely demographics, care handler, diagnosis and treatment; administrative and financial; and laboratory and Para clinic.

Assessment of the HIS Software used in these three hospitals indicated low HISRA for entire data elements (58.5%). It was even lower (47%) when considering research and educational data elements. The diagnosis and treatment data group had the poorest HISRA (less than 45%) among the five data groups, while HISRA for demographics, laboratory and Para clinic; and administrative and financial data groups was over 90%. Poor HISRA observed for the diagnosis and treatment data group was due to the lack of corresponding data elements in the HIS software to capture some essential data such as physical examinations; detailed nurse notes; vital signs control; fluid absorption/excretion; pre-operation; post-operation, and anesthesia care, and their timelines. HIS software flaw also has increased inaccuracy of the diagnosis and treatment data group due to the lack of data validity checks in open-text fields.

For care handler; and diagnostic and treatment data, although the accuracy was relatively good (over 80% in all hospitals), incompleteness reduced the overall data quality to less than 70% for both entire data; and research and educational data. We observed that policies and regulations flaw has led to reduced completeness of diagnostic and treatment data in the three hospitals because caregivers are not required to record medical history, disease progress received consultations, and physician orders completely in electronic records. Poor policies and regulations also resulted in reduced completeness of the care handler group because clinical staff did not have to capture such information within the HIS. In the care handler group, inaccuracy commonly resulted from the wrong timestamps. Because the timestamp was created automatically by the computer and for a late data entry, which was not done at the point of medical action, it reflects the time of computerized data entry, not the real-time of the medical procedure. Moreover, EMRs sometimes included the user who entered the data instead of the doer of medical procedures.

According to our observations, the quality of laboratory and para clinic data in hospital B was the highest (over 90%). It was relatively good (over 80%) in hospital A, and unexpectedly poor in hospital C. This happened because, in the hospital C, a laboratory or para clinic report is recorded in both electronic and paper records, provided that it was done by the hospital's laboratory or para clinic section. Otherwise, it is held only in paper records. Whereas, in this hospital, it is common to refer patients to outside laboratories to do some physician-ordered tests. Similarly, the three hospitals, capture the medicine administration in the patient's EMR when the ordered medicine has been provided by the hospital's pharmacy. Otherwise, it was recorded only in the paper charts as this procedure flaw has increased inconsistency between paper and electronic records and has led to low completeness of laboratory and Para clinic data in hospital C and low completeness of diagnosis and treatment data in all three hospitals.

Several studies investigated the quality of electronic medical data used for research purposes; they employed various assessment strategies to report data quality. We found studies that referred to “data availability” as “completeness” and assessed it by three major strategies, (i) comparing with a gold standard, (ii) checking the presence or absence of data value, and (iii) comparing with data from other data sources.

We also found studies that defined “agreement among data elements” as “concordance, consistency, validity, correctness or accuracy”. Considering the publications that we found, data agreement was assessed by two main strategies, including (i) Data Element Agreement, comparing the data with those within the EMR, and (ii) comparing the data with those from other sources that may be a gold standard. Other used data sources include paper records (11–18) patient-reported data (19–21) or physician-reported data (22). Some studies reported the overall completeness and accuracy of the electronic data, comparing paper records as the “gold standard” (11–14, 23). Some others compared electronic medical data to paper records as the “data source”(15–17).

Compared to the mentioned studies, our study had a higher level of granularity of the elements measured and benefited from extensive assessment. Although most studies assessed data elements that serve the purpose of doing a determined study in the future (diagnosis and procedure codes related to a specific disease, for instance) (11–14, 16, 17), our study did not focus on the suitability of data elements for determining research. We tried to identify and assess essential data for research and education. We also expanded the assessment to entire recorded data (even financial ones) to investigate how to improve HIS to collect effective records. Our definitions of completeness, and accuracy, and the protocol we used to measure them were consistent with some previous studies. However, it was quite different from that employed by Whitelaw et al. (14) and Logan et al. (21) and also was somewhat different from the definition of Prins et al. (13).

In light of our findings, the following suggestions are offered to improve the HIS software used in these hospitals and as instructions to be given to the medical staff:

Given the low HISRA of the software, particularly in terms of diagnosis and treatment data, the HIS used in these hospitals needs to improve to record physical examinations, pre-operation, post-operation, anesthesia care, detailed nurse notes, vital signs, fluid absorption and excretion, and their time trends. Additionally, in these hospitals, a great amount of data are entered in an open text and non-structural form. By adding data-entry fields, software capabilities can facilitate input validation checking, which may increase the completeness and accuracy of the recording process. Considering the incompleteness of the care handler data group, the medical staff should be instructed to record the doers of the medical action. Moreover, digital signature and audit logging should be implemented to assure care handler data accuracy.

Hospitals should set strict rules assuring the medical staff records the patient's history, physician's orders, disease progress notes, and consultations. HIS also must be enabled to record prescriptions and medications together with clinical information received from other medical centers outside the hospital to track medication and refill histories. This trend will certainly increase the completeness of the diagnosis and treatment; and laboratory and Para clinic data.

Since we frequently observed inaccuracy in date and time in the diagnosis and treatment data group, HIS must be redesigned to record the date and time for each entry based on the actual date and time. HIS must also provide the documenter with the possibility of manually entering the date and time of late entries.

If it is necessary to have both electronic and paper medical records in parallel, it is recommended to omit the hand-written paper records and print the computer-generated EMR. This procedure ensures legibility, completeness, validity, and precision of the paper record by utilizing software controls such as “input masks”. It also increases the concordance between electronic and paper records.

Studies that use the “Data Source Agreement” strategy to assess the quality of electronic data prefer to compare data with a valid and reliable external data source. We used patients' paper records as the assessment standard even though they might have been illegible, incomplete, or unreliable; they were the only available documentation usually used in addition to electronic data by medical care providers in the mentioned hospitals. Thus, the absence of a reliable external data source formed a limitation of the present study. Furthermore, the result was not influenced by the transformation of the electronic record or the paper record, or both (because data values often had been recorded in the form of open text). However, accuracy assessment was influenced by the personal impression of the investigators as well as the performance of the persons who entered the data in to the EMRs.

### Future work

As a possible future work, one could investigate the degree to which the data deviate from a truly “random sample” of the patients' data. For example, if normal laboratory values are more likely to be in the computer and abnormal values are more likely to be on paper, this could have a pronounced impact, and bias, on research that attempts to use only one or the other.

## Conclusions

The findings demonstrated that the HISRA of the system was not good in general, and it was poor for diagnosis and treatment data in particular. It also showed the low quality of registering the care handler; and diagnosis and treatment data groups.

The high quality of administrative and financial data groups imply that in the three hospitals, the development of the software and data entry policies focus on managerial, administrative, and financial applications rather than potential academic and clinical applications. Thereby, it is suggested that the clinical and diagnostic aspects of those EMRs should be addressed. Easier data entry methods, better training of personnel, and changes in the HIS software can probably solve many obstacles. Furthermore, regulations and policies need to be revised to serve the data quality improvement.

## Data Availability

The data analyzed in this study is subject to the following licenses/restrictions: The data for this study was obtained from databases in three hospitals that belong to Mashhad University of Medical sciences. Both forms of electronic and paper records are protected severely from general access by confidentiality and privacy regulations, and in addition to the care providers, researchers affiliated with the University have access to anonymized records for a definite time. None of the authors had access to any identifying patient information. The data were anonymized at the point of extraction from the systems and paper forms. The researcher signed a commitment to maintaining the confidentiality of information which do not allow them to keep, disclose or reuse the information, for another study, for example, or other purposes after the end of the study. Requests to access these datasets should be directed to the corresponding author.
